# Association Study of Serotonin Transporter SLC6A4 Gene with Chinese Han Irritable Bowel Syndrome

**DOI:** 10.1371/journal.pone.0084414

**Published:** 2014-01-02

**Authors:** Jing Yuan, ChuanYuan Kang, Min Wang, Qiang Wang, PeiKai Li, Hua Liu, Yu Hou, Ping Su, Fan Yang, YuJun Wei, JianZhong Yang

**Affiliations:** 1 Clinical Psychology Department, No. 2 People's Hospital of Yunnan Province, Kunming, China; 2 Psychiatry Department, The First Affiliated Hospital of Kunming Medical University, Kunming, China; 3 Gastroenterology Department, No. 2 People's Hospital of Yunnan Province, Kunming, China; 4 Department of Laboratory, The First Affiliated Hospital of Kunming Medical University, Kunming, China; Baylor College of Medicine, United States of America

## Abstract

**Objective:**

Irritable bowel syndrome (IBS) is a common clinical gastrointestinal dysfunction disorders. 5-sertonon (5-hydroxytryptamine, 5-HT) is a very important neurotransmitter, which is involved in gastrointestinal motion and sensation. Solute carrier family 6 member 4 (SLC6A4) gene encode serotonin transporter (SERT) which function is to rapidly reuptake the most of 5-HT. Therefore, it is needed to explore the association between SLC6A4 gene polymorphisms and IBS.

**Methods:**

119 patients and 238 healthy controls were administrated to detect the SLC6A4 gene polymorphisms including 5-HT-transporter-gene-linked polymorphic region (5-HTTLPR), variable number of tandem repeats (VNTRs) and three selected tag Single Nucleotide Polymorphisms (SNPs) rs1042173, rs3794808, rs2020936 by using polymerase chain reaction (PCR) and TaqMan® SNP Genotyping.

**Results:**

There were significant difference for 5-HTTLPR between IBS and control groups (X2 = 106.168, P<0.0001). In control group, genotypes were mainly L/L (58.4%), however, the genotypes in IBS were S/S (37.8%). The significant difference was shown in D-IBS subjects when compared to the controls (X^2^ = 50.850, P<0.0001) for 5-HTTLPR. For STin2 VNTR, rs1042173, rs3794808, and rs2020936 polymorphisms, there were no any significant differences between IBS and control groups. There were no statistical significantly haplotypes for 5-HTTLPR, VNTRs and the three SNPs between IBS and controls.

**Conclusion:**

The S allele in 5-HTTLPR was a susceptible allele with Chinese Han IBS, but other associations of VNTRs, three selected Tag SNPs and positive haplotype with IBS were not found. It is indicated that much research are needed to study the relationship between other polymorphisms in SLC6A4 gene and IBS.

## Introduction

Irritable bowel syndrome (IBS) is a chronic gastrointestinal disorder with that affects 10–15% of the population [1]. Studies have shown that genes may play an important role in IBS. Genetic factors may be directly linked to gastrointestinal sensory and motor functions or cause initiation of the modifications underlying the symptoms in the presence of exogenous factors [2].

At the gastrointestinal level, 5-HT acts as a paracrine signalling molecule and as a transmitter released by serotonergic interneurons. Serotonin activates at least five types of receptors, influencing intestinal peristalsis, secretion and signalling in the brain-gut axis. A high level of 5-HT may result from exaggerated synthesis, excessive release, or inadequate uptake and inactivation. Modifications in the serotonin transporter, responsible for removing 5-HT from the interstitial space and terminating its action, may also contribute to gastrointestinal motility troubles [3]. So 5-HT is thought to play a key role in the pathophysiology of IBS [4], [5].

Recent studies have demonstrated the importance of polymorphisms in the promoter region of the serotonin reuptake transporter (SERT) gene for motility disorders [3]. Solute carrier family 6 member 4 (SLC6A4) gene encoding (SERT resides on chromosome 17q11.1–17q12 in the human genome, spanning more than 30 kb, consisting of 14 exons and encoding a 630-amino acid protein. A functional polymorphism, an insertion or deletion of 44 base pairs in the 5-HT-transporter-gene-linked polymorphic region (5-HTTLPR), was first reported in 1996 by Heils [6]. Lately, there were studies supported that the SERT polymorphism or a polymorphism in linkage disequilibrium with the SERT polymorphism might play a role in the development of IBS [3], [7]. A second SERT polymorphism, variable numbe of tandem repeats (VNTR) STin2, located in intron 2 and consisting of a variable number (usually 9, 10, or 12) of nearly identical 17-bp segments, had been found to be associated with IBS in one study, with the 10/12 genotype more frequent in Chinese patients than in controls [8]. Most authors, however, had found no association between STin2 VNTRs and IBS [3], [9], [10].

Though 5-HTTLPR and STin2 VNTRs were implicated in the pathogenesis in Chinese IBS patients [8], [10], and significant heterogeneity was always present in some genetic studies [9], [11]. Therefore in this study, we hypothesized that there were positive associations of SERT polymorphisms with Chinese IBS. The aim of study is to investigate association of the SLC6A4 genetic polymorphisms including 5-HTTLPR, STin2 VNTRs and three selected tag SNPs (rs1042173, rs3794808, rs2020936) with IBS, then to provide the evidence for the genetic pathogenesis of IBS.

## Materials and Methods

### Ethics Statements

All the subjects were Chinese of Han descent. IBS patients and healthy comparison had no any biological relationship. All subjects gave written informed consent to participate in the research. The study was approved by the Ethics Committee of the No. 2 People's Hospital of Yunnan Province.

### Subjects

119 Patients were recruited by either referral from medical clinics at the No. 2 People's Hospital of Yunnan Province or through advertisements displayed in the hospital. These patients were then further screened by two gastroenterologists with more than 20 years of specialist experience in the research team and were considered eligible to join the study, if they satisfied the inclusion criteria: they had to be the age between 18 and 65 years old, and had to fulfill the Rome III consensus criteria for the diagnosis of IBS. Patients had to undergo an upper gastrointestinal endoscope and colonoscopy, if they had not undergone within the preceding 3–6 months, to exclude the presence of any organic disease. Those found to have evidence of H. pylori infection by urea breath test (UBT) were excluded. Among these patients, there were 80 diarrhoea predominant IBS (D-IBS), 21 constipation predominant IBS (C-IBS), and 18 alternating diarrhoea and constipation (M-IBS).

238 healthy comparison subjects (male 139 and female 99) from regular physical examination who were without IBS were recruited from the same area and were matched for age and gender to the IBS subjects. Exclusion criteria included celiac disease, diabetes mellitus, major abdominal surgery, endocrine, central nervous system, or severe psychiatric disorders as assessed by history taking, physical examination, laboratory tests.

Peripheral blood samples were obtained from all subjects and genomic DNA was extracted using the phenol–chloroform method.

### Genotype

By using software of Haploview 4.2 version (http://hapmap.org/), with minimum allele frequency >10%, D' = 1, r^2^>0.8, we selected three Tag single nucleotide polymorphisms (SNPs) (rs1042173, rs3794808, rs2020936) which represented the three blocks in this gene from the linkage disequilibrium map. The Tag SNPs were then genotyped by using TaqMan® SNP Genotyping Assays on an 7300RT PCR SYSTEM (Applied Biosystems, Foster City, Calif., USA) according to standard protocols (C_7473190_10, C_27488353_10, C_11414119_10).

We also replicated 5-HTTLPR [12] and STin2 VNTR [13] in this study.

### Statistical analysis

Statistical analysis of association was performed using SPSS 17.0 software. Deviation from Hardy-Weinberg equilibrium and case- control study were tested using the χ2 test for goodness of fit and χ2 test for dependence, respectively. Linkage disequilibrium (LD) and Case-control haplotype analysis were conduct by SHEsis software (http://analysis.bio-x.cn/myAnalysis.php) [14]. Linkage disequilibrium (LD) was tested using the χ2 test, and D' and r2 values were made the index in the authorization of LD. Case-control haplotype analysis was performed by the permutation method, and permutation p-values and Odds ratios were calculated based on 100,000 replications. All genetic statistical analysis was verified by software Haploview. The statistic power of this sample was calculated by the GPower 3.1.2 program [15], [16].

## Results

### 1. Demographic characteristics

Of the patients, there were 67 (56%) male and 52 (44%) female, mean age 43.96±14.70 years. The mean duration of illness was 72.93±7.71 months. The average educational level was 11.05±3.99 years.

The mean age of healthy subjects was 45.12±8.68 years, and the mean duration of education was 13.76±3.06 years.

### 2. Genotype and allele frequencies of five polymorphisms between IBS patients and healthy controls

Genotype distributions for the 5-HTTLPR, STin2 VNTR, rs1042173, rs3794808, and rs2020936 polymorphisms were checked for deviation from Hardy–Weinberg equilibrium and no deviation was observed (5- HTTLPR χ^2^ = 1.3, P>0.05; STin2 VNTR χ^2^ = 1.5, P>0.05; rs1042173 χ^2^ = 0.12, P>0.05; rs3794808 χ^2^ = 0.17, P>0.05; rs2020936 χ^2^ = 0.37, P>0.05). Allele frequencies were shown in Table 1.

**Table 1:** Genotype distributions and allele frequencies five polymorphisms between IBS patients and healthy controls.

Polymorphisms	Genotypes Distributions n (%)					Allele	Allele Frequency n (%) IBS Controls	
**5-HTTLPR**	S/S	S/L	L/L	L/XL	S/XL	S	121 (50.8)	53 (11.1)
IBS	45 (37.8)	31 (26.1)	30 (25.2)	13 (10.9)	0(0)	L	104 (43.7)	352 (74)
Controls	20 (8.4)	8 (3.4)	139 (58.4)	66 (27.7)	5(2.1)	XL	13 (5.5)	71 (14.9)
**STin2 VNTR**	9/11	10/10	11/11	Other (10/11, 10/12, 12/12)		STin2.9	16 (6.7)	23 (4.8)
IBS	14 (11.8)	4 (3.4)	99 (83.2)	2 (1.6)		STin2.10	9 (3.8)	26 (5.5)
Controls	23 (9.7)	11 (4.6)	200 (84.0)	4 (1.7)		STin2.11	213 (89.5)	425 (89.3)
						STin2.12	0 (0)	2 (0.4)
**rs1042173**	GG	GT	TT					
IBS	77 (64.7)	34 (28.6)	8 (6.7)			G	188 (0.790)	378 (0.794)
Controls	153 (64.3)	72 (30.3)	13 (5.4)			T	50 (0.210)	98 (0.206)
**rs3794808**	AA	AG	GG					
IBS	81 (68.1)	9 (7.6)	29 (24.3)			A	171 (71.8)	322 (67.6)
Controls	154 (64.7)	14 (5.9)	70 (29.4)			G	67 (28.2)	154 (32.4)
**rs2020936**	CC	CT	TT					
IBS	5 (4.2)	25 (21.0)	89 (74.8)			C	35 (0.147)	77 (0.162)
Controls	8 (3.4)	61 (25.6)	169 (71.0)			T	203 (0.853)	399 (0.838)

Comparing genotype distributions between IBS and controls (Table 1) showed that there were significant differences for 5-HTTLPR between IBS and control groups (X^2^ = 106.168, P<0.0001). In control group, genotypes were mainly L/L (58.4%), however, the genotypes in IBS were S/S (37.8%). There was a trend towards association of the 5-HTTLPR allele with IBS (X^2^ = 137.437, P<0.001). For IBS subjects, there were 50.8% S allele, 43.7% L allele, and 5.5% XL allele. However, for controls there were 11.1% S allele, 74% L allele, and 14.9% XL allele. The same significant difference was shown in D-IBS subjects which there were 58.8% S allele, 37.5% L allele, and 3.7% XL allele when compared to the healthy controls (X^2^ = 50.850, P<0.0001).

For STin2 VNTR, rs1042173, rs3794808, and rs2020936 polymorphisms, there were no any significant differences between IBS and control groups, neither in genotypes distributions nor allele frequencies.

### 3. Haplotype frequencies of five polymorphisms between IBS patients and healthy controls

We calculated pairwise linkage disequilibrium between the investigated polymorphisms, using the SHEsis program. Table 2 showed rs1042173 and rs3794808 was in strong linkage disequilibrium (D' = 0.938), but other LDs composed by other polymorphisms were relative weak. Haplotype analysis with rs1042173 and rs3794808 did not showed any significant difference between the patients and the controls.

**Table 2:** Linkage disequilibrium.

	VNTR	Rs1042173	Rs3794808	Rs2020936
5-HTT	0.100	0.156	0.163	0.115
VNTR	-	0.419	0.405	0.462
Rs1042173	-	-	0.938	0.641
Rs3794808	-	-	-	0.593

When αwas set under 0.05, the power analysis showed the statistic power was 91.3%.

## Discussion

In a meta-analysis [17], there is no association between the genetic polymorphism in the SERT-P gene and IBS, however, in Asians, majority of patients were homozygous for the short allele (64%). In another meta-analysis [18], when stratified the selected studies by the participants' region or population, a reduced effect of the polymorphism on IBS risk in American and Asian studies.was found.

In the present study, an association between 5-HTTLPR polymorphism and IBS had been observed, especially in the D-IBS patients. S allele was present mostly in IBS patients (50.8%), and L allele was predominant in the controls (74%). The SERT protein is responsible for reuptake of 5-HT in serotonergic nerves and mucosa of bowel, and is a factor that determines 5-HT activity [4]. In a lymphoblast cell line, s/s genotype at promoter polymorphic site of SERT gene was associated with lower transcriptional efficiency, resulting in lower SERT expression and therefore lower cellular uptake of 5-HT [19]. In animal models, SLC6A4 knockout mice had diarrhoea, which was associated with faster colonic motility that resulted in increased excretion of water in stool [20]. Studies showed that 5-HT concentration was higher in D-IBS patients after meals than healthy controls [21]. These observations could explain our results showing SLC6A4 s/s genotype to be associated with D-IBS which were approved by several researches in different races [3], [9], [12]. The association of D-IBS with the SLC6A4 s/s genotype was also supported by other studies demonstrating changes in postprandial 5-HT levels in platelet-depleted plasma [21], [22]. However, our results were different from precious Chinese studies, in which genotypes of SS+IS were associated with reduced risk of IBS. As we know, IBS pathogenesis is considered to be multifactorial, and single genetic variant is usually insufficient to predict the risk of this disease. One important characteristic of this gene polymorphism is that their incidence may vary substantially between different populations and/or ethnicities [18].

For VNTR STin2 and three selected Tag SNPs which represented three blocks in the gene and were the first report in IBS study, there were no significantly positive associations found. The haplotype analysis composed by the five polymorphisms still did not show any positive association with IBS. For VNTR STin2, only one study found to be associated with IBS, with the 10/12 genotype more frequent in Chinese patients than in controls [8]. In our study, 83.2% subjects were 11/11 genotype, apparently different from Wang's result. One of the reasons for the variety was the heterogeneous pathogenesis. IBS may be heterogeneous in different populations as it is a syndrome diagnosed using symptom-based criteria, its prevalence being variable in different countries with different ethnicity [23], [24], [25]. Such heterogeneity in phenotypes may also partly explain the result of genetic studies.

Several limitations to this study should be noted. First, the sample size of the present study is still small for genotypic analysis though the sample number in this present study was larger than Wang's study [8], so the results cannot be applied to the general population and should be interpreted carefully. Second, the study population included all of the subtypes of IBS, which might have contributed to clinical heterogeneity; the subtypes may have acted as potential confounders of the investigated association.

In conclusion, the S/S genotype was the common type in IBS group for 5-HTTLPR in SLC6A4 gene, but the frequent genotype in healthy controls was L/L. It was suggested the S allele in 5-HTTLPR is a susceptible allele with IBS, but we did not find the association between the polymorphisms of VNTRs, the three selected Tag SNPs(rs3794808, rs2020936, rs1042173)and positive haplotype with IBS. It is indicated that much research are needed to study the relationship between other polymorphisms in SLC6A4 gene and IBS. Moreover, confirmation of the association with IBS in a larger sample of subjects is clearly needed, along with further studies on SERT function in IBS and the effects of genetic variants on SERT function in gut cells.
